# Prognostic factors and benefit populations of ovarian function suppression in premenopausal HR+/HER2+ early‐stage breast cancer patients who received trastuzumab: Evidence from a real‐world study with long‐term follow‐up

**DOI:** 10.1111/1759-7714.15211

**Published:** 2024-01-07

**Authors:** Jie Ju, Song‐Lin Gao, Jia‐Yu Wang, Die Sang, Yi‐Kun Kang, Xue Wang, Jian Yue, You Shuai, Yi‐Xin Qi, Peng Yuan

**Affiliations:** ^1^ Department of Medical Oncology, National Cancer Center/National Clinical Research Center for Cancer/Cancer Hospital Chinese Academy of Medical Sciences and Peking Union Medical College Beijing China; ^2^ Key Laboratory of Carcinogenesis and Translational Research (Ministry of Education/Beijing), The VIPII Gastrointestinal Cancer Division of Medical Department Peking University Cancer Hospital and Institute Beijing China; ^3^ Department of Medical Oncology Beijing Sanhuan Cancer Hospital Beijing China; ^4^ Department of Medical Oncology, Beijing Hospital, National Center of Gerontology, Institute of Geriatric Medicine Chinese Academy of Medical Sciences Beijing China; ^5^ Department of VIP Medical Services, National Cancer Center/National Clinical Research Center for Cancer/Cancer Hospital Chinese Academy of Medical Sciences and Peking Union Medical College Beijing China; ^6^ Department of Breast Center The Fourth Hospital of Hebei Medical University Shi Jiazhuang China

**Keywords:** breast cancer, hormone receptor, human epidermal growth factor receptor 2, ovarian function suppression, trastuzumab

## Abstract

**Background:**

Hormone receptor‐positive (HR+)/human epidermal growth factor receptor 2‐positive (HER2+) breast cancer exhibits considerable heterogeneity, and it is of great interest whether patients with premenopausal HR+/HER2+ breast cancer treated with trastuzumab can benefit from ovarian function suppression (OFS) therapy similarly to HR+/HER2‐ breast cancer. Here, we conducted a real‐world study in this population to identify both who would derive substantial benefits from the addition of OFS and clinicopathological factors with potential prognostic value.

**Methods:**

Multicenter data from 253 premenopausal patients with HR+/HER2+ early‐stage breast cancer who received trastuzumab from October 2009 to October 2018 were retrospectively included. The Kaplan–Meier method was used for survival analysis, while the log‐rank test was used to compare the survival rates. Univariate and multifactor Cox regression analyses were performed to analyze the independent risk factors affecting invasive disease‐free survival (IDFS).

**Results:**

After a median follow‐up of 98.50 months, compared with tamoxifen/toremifene alone, tamoxifen/toremifene/aromatase inhibitors plus OFS demonstrated significant benefits in the overall study population (HR = 0.289, 95% CI: 0.100–0.835, *p* = 0.022, 8‐year IDFS rate: 90.78% vs. 95.54%), especially in the lymph node‐positive subgroup and age ≤40 years subgroup. Age ≤40 years, histological grade >2, lymph node involvement, PR ≤50%, and tamoxifen alone were independent prognostic factors.

**Conclusions:**

For premenopausal HR+ breast cancer patients, HER2 positivity alone is an indication for the addition of OFS in adjuvant endocrine therapy. Age, histological grade, lymph node status, the expression of PR, and OFS treatment were independent prognostic factors in this population.

## Graphical abstract

In the context of the widespread use of trastuzumab, the efficacy of OFS in premenopausal patients with HR+/HER2+ early‐stage breast cancer is still controversial. We conducted a retrospective review of 253 premenopausal patients with HR+/HER2+ early‐stage breast cancer treated with trastuzumab, and found that the addition of OFS had a significant benefit for the overall population. Age, histological grade, lymph node status, PR status, and OFS treatment were independent prognostic factors in this population.

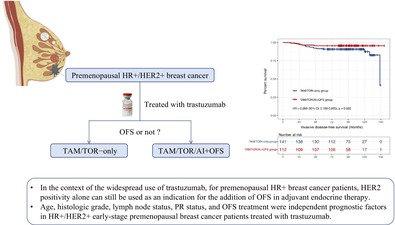

## INTRODUCTION

The latest global cancer burden data for 2020 has shown that breast cancer has become the most common cancer, superseding lung cancer for the first time.^1^ In China, breast cancer patients are characterized by a large base, rapid increase, and young onset age. According to statistics, up to 60% of patients are premenopausal at the time of diagnosis, which is far more than in the Western population.^2^ Up to 60%–80% of breast cancers are estrogen receptor‐positive (ER+), and the suppression of estrogen produced by the ovaries reduces recurrence in premenopausal women with hormone receptor HR+ breast cancer. Based on several key phase III clinical studies, such as the SOFT and ASTRRA studies, authoritative guidelines have recommended the addition of OFS for premenopausal patients with HR+ breast cancer who are prone to recurrence or metastasis.^3^, ^4^ However, the vast majority of patients in these studies were HR+/HER2−, and approximately only 12.3%–13.7% of patients were HR+/HER2+ furthermore, due to the limitations of the time, only some of these HR+/HER2+ patients were treated with trastuzumab. This has sparked a debate about the value of OFS in the treatment of trastuzumab‐treated premenopausal HR+/HER2+ early‐stage breast cancer.

On the one hand, unlike other subtypes of breast cancer, HR+/HER2+ breast cancer is endowed with distinct molecular biological characteristics, therapeutic responses and drug resistance mechanisms due to the complex interaction between HR and the HER2 signaling pathway.^5^, ^6^ According to our previous studies, the expression levels of HER2 and ER in HR+/HER2+ breast cancer impact patient prognosis, and DFS is significantly shortened in patients with higher HER2 expression (*p* = 0.046; 5‐year DFS rate: 67% vs. 91%).^7^ On the other hand, under natural conditions, HER2 promotes the invasion and metastasis of tumor cells by promoting cell division, which is closely related to poor prognosis. However, the widespread utilization of trastuzumab, an anti‐HER2 targeting agent, has greatly improved the prognosis of HER2+ breast cancer patients. In the eighth edition of the American Joint Committee on Cancer (AJCC) staging manual, HER2 is no longer associated with poor prognosis.^8^ The improvement in the natural course of disease with trastuzumab should not be ignored. In summary, the treatment of HR+/HER2+ breast cancer is not a simple combination of multiple treatments, and it is risky to directly apply research findings derived from HR+/HER2− patients to HR+/HER2+ patients.

We conducted this retrospective real‐world study in trastuzumab‐treated premenopausal early‐stage HR+/HER2+ breast cancer patients from multiple centers with the objectives of (1) identifying the real beneficiaries of OFS treatment in these patients and (2) exploring the clinicopathological factors affecting the prognosis of HR+/HER2+ patients.

## METHODS

### Study population

This retrospective study included 253 patients with HR+/HER2+ breast cancer from October 2009 to October 2018 in the Cancer Hospital Chinese Academy of Medical Sciences, the Beijing Sanhuan Cancer Hospital, and the Fourth Hospital of Hebei Medical University. The inclusion criteria were as follows: (a) women who remained premenopausal at the time of surgery, (b) age ≤60 years old when diagnosed, (c) stage I–III invasive breast cancer, (d) HR‐positive (defined as ER ≥10% and/or PR ≥10%) and HER2‐positive, (e) received radical breast cancer surgery and 1 year of adjuvant trastuzumab without neoadjuvant therapy and (f) OFS use, if applicable, lasting at least 2 years. The exclusion criteria were as follows: (a) patients with other malignant tumor, (b) patients with serious complications of heart, liver or kidney, (c) patients with at least 3 months of survival and (d) patients with incomplete clinicopathological features, treatment or follow‐up information. Patients were allocated to two groups according to the endocrine treatment regimens: the tamoxifen/toremifene group (TAM/TOR‐only group) and the tamoxifen/toremifene/aromatase inhibitors plus OFS group (TAM/TOR/AI+OFS group). OFS drugs included goserelin and leuprorelin. AIs included exemestane, anastrozole and letrozole.

### Data collection and outcomes

We retrieved data from the hospital information system. The following data were extracted from pathological reports: tumor size, lymph node status, lymphovascular invasion (LVI), histological type, histological grade, and expression of ER, PR, HER2, and Ki67. We also collected information regarding age at diagnosis, menopausal status, date of radical breast cancer surgery, type of surgery, and adjuvant treatment information from inpatient and outpatient medical records. We followed up patients for survival via telephone or email.

The expression of ER, PR, HER2, and Ki67 was assessed by immunohistochemistry (IHC) staining. The HR‐positive percentage was defined as the highest percentage of ER‐positive or PR‐positive cells. HER2 positivity was defined as 3+ according to IHC analysis or amplification confirmed by fluorescence in situ hybridization (FISH).

The endpoint of the study was invasive disease‐free survival (IDFS), which was defined as the time from radical surgery to one of the following events: ipsilateral invasive breast tumor recurrence, regional invasive breast cancer recurrence, distant recurrence, contralateral invasive breast cancer, second primary nonbreast invasive cancer, or death by any cause.

### Statistical analysis

Statistical analysis was performed using SPSS 23.0 (IBM, Chicago, IL, USA) and R software (version 4.1.1, http://www.r-project.org). The categorical variables are expressed as numbers and percentages, with the *χ* ^2^‐test or Fisher's exact test for between‐group comparisons, whereas the continuous variables are presented as the mean and standard deviation, with Student's *t*‐test or Kruskal–Wallis H‐test for between‐group comparisons. Survival analysis was carried out based on the Kaplan–Meier method, and the forest plot of subgroup analysis was drawn based on univariate Cox regression. Univariate Cox regression analyses were performed to identify significant prognostic factors, and the variables with Wald's *p* < 0.2 were selected for subsequent multivariate analyses. Based on the results of Cox multivariate analysis, a prognostic nomogram was generated for IDFS. A two‐tailed *p*‐value of 0.05 or less was considered statistically significant.

## RESULTS

### Baseline characteristics of the overall population

Overall, 253 patients with HR+/HER2+ early‐stage breast cancer who received trastuzumab were included in the study. The clinicopathological characteristics are shown in Table 1. All patients received radical surgery, and 251 received adjuvant chemotherapy. All patients received adjuvant endocrine therapy, including 112 patients who received OFS treatment. Of the 137 patients with lymph node‐positive breast cancer, 80.3% (110/137) received adjuvant radiotherapy. Of the 116 patients with lymph node‐negative breast cancer, 41.4% (48/116) received adjuvant radiotherapy.

**TABLE 1:** Baseline characteristics of 253 premenopausal HR+/HER2+ patients.

Characteristics	Cases	Percentage
Age/year		
≤40	103	40.7%
>40	150	59.3%
Histological grade		
I–II	133	52.6%
III	120	47.4%
Tumor size/cm		
≤2	118	46.6%
>2	135	53.4%
Lymph node status		
Negative	116	45.8%
Positive	137	54.2%
Ki67 index		
<30%	59	23.3%
≥30%	194	76.7%
ER status		
<50%	52	20.6%
≥50%	201	79.4%
PR status		
<50%	99	39.1%
≥50%	154	60.9%
LVI		
Negative	145	57.3%
Positive	86	34.0%
Unknown	22	8.7%
Surgery		
Lumpectomy	60	23.7%
Mastectomy	193	76.3%
Chemotherapy regimen		
Nonanthracycline	68	26.9%
Anthracycline‐based	183	72.3%
Unknown	2	0.8%
Endocrine therapy		
TAM/TOR‐only	141	55.7%
TAM/TOR/AI+OFS	112	44.3%
Radiation therapy		
No	83	32.8%
Yes	158	62.5%
Unkonwn	12	4.7%

Abbreviations: ER, estrogen receptor; HER2, human epidermal growth factor receptor 2; LVI, lymphovascular invasion; OFS, ovarian function suppression; PR, progesterone receptor; TAM, tamoxifen; TOR, toremifene.

### Survival analysis

**FIGURE 1:**
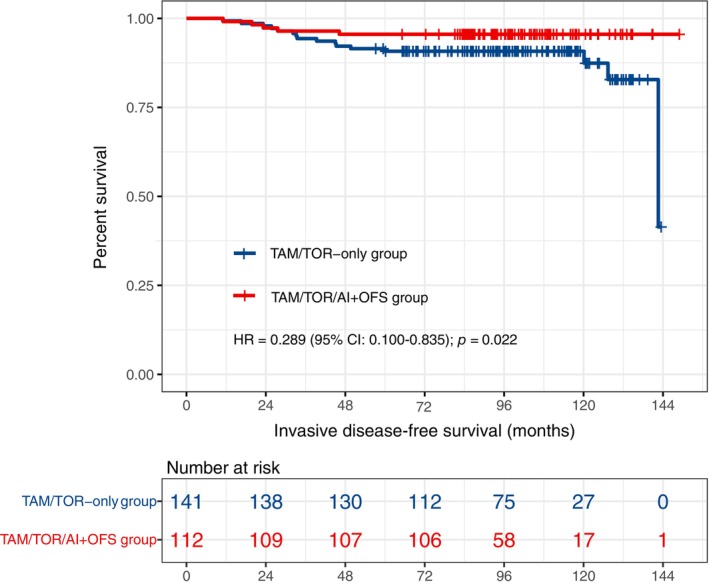
Kaplan–Meier curve of IDFS for patients in TAM/TOR‐only versus TAM/TOR/AI+OFS group. AI, aromatase inhibitor; CI, confidence interval; HR, hazard ratio; IDFS, invasive disease‐free survival; OFS, ovarian function suppression; TAM, tamoxifen; TOR, toremifene.

**FIGURE 2:**
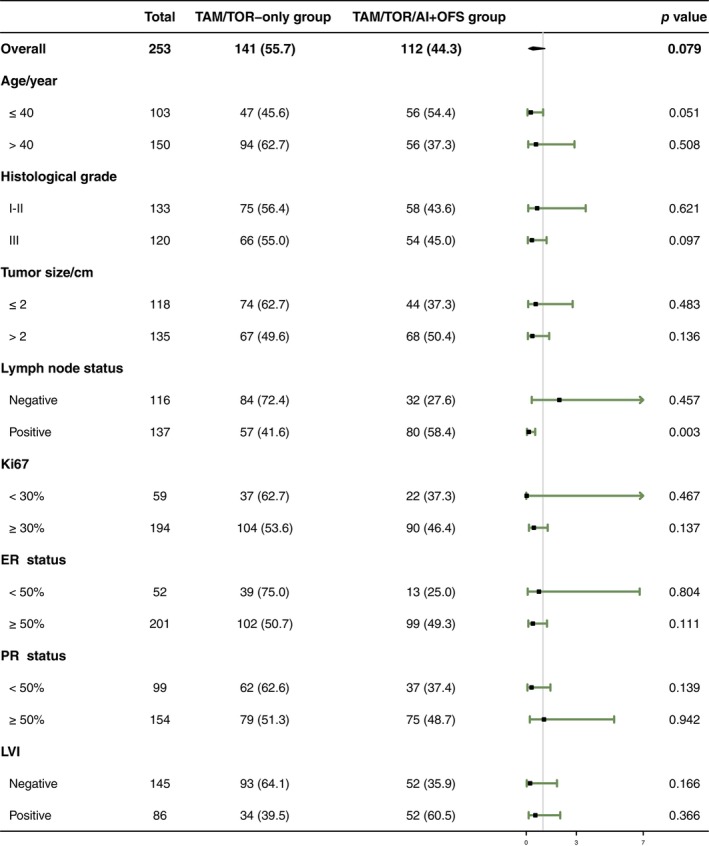
Forest plot demonstrating the HR of different subgroups. AI, aromatase inhibitor; ER, estrogen receptor; LVI, lymphovascular invasion; OFS, ovarian function suppression; PR, progesterone receptor; TAM, tamoxifen; TOR, toremifene.

Until May 20, 2023, the median follow‐up time was 8.21 years. In the TAM/TOR‐only group, 16 patients had relapse and metastasis. Meanwhile, in the TAM/TOR/AI+OFS group, patients had relapse and metastasis. Multivariate Cox regression significantly differed between the two groups (HR = 0.289; 95% CI: 0.100–0.835; *p* = 0.022; 8‐year IDFS rate: 90.78% vs. 95.54%, Figure 1). Further subgroup analysis based on univariate Cox regression showed more significant benefits in the node‐positive subgroup and the age ≤40 years subgroup (Figure 2), and the specific results of subgroup analysis are presented in Table S1.

### Analysis of prognostic factors

**FIGURE 3:**
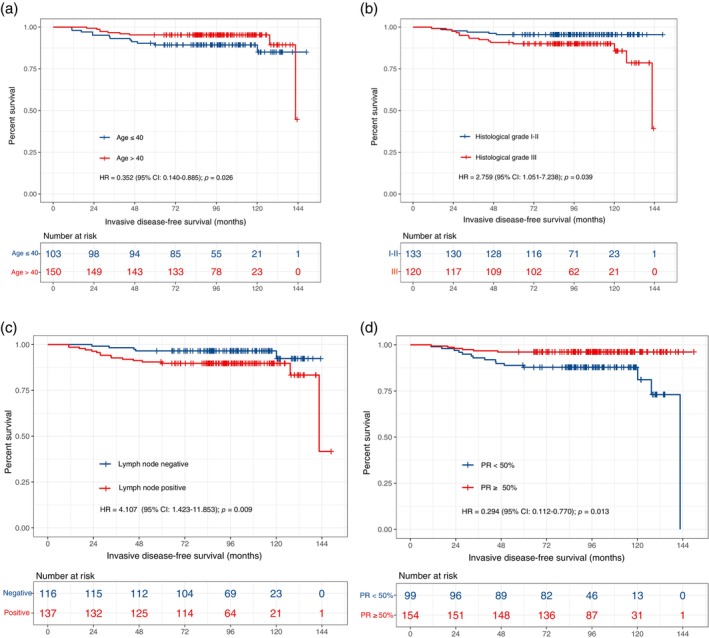
Kaplan–Meier curves of IDFS of patients in different subgroups. (a) Kaplan–Meier curve of IDFS for patients in age ≤40 versus age >40 group. (b) Kaplan–Meier curve of IDFS for patients in histological grade I–II versus histological grade III group. (c) Kaplan–Meier curve of IDFS for patients in lymph node‐negative versus lymph node‐positive group. (d) Kaplan–Meier curve of IDFS for patients in PR <50% versus PR ≥50% group. CI, confidence interval; IDFS, invasive disease‐free survival; HR, hazard ratio; IDFS, invasive disease‐free survival; PR, progesterone receptor.

**FIGURE 4:**
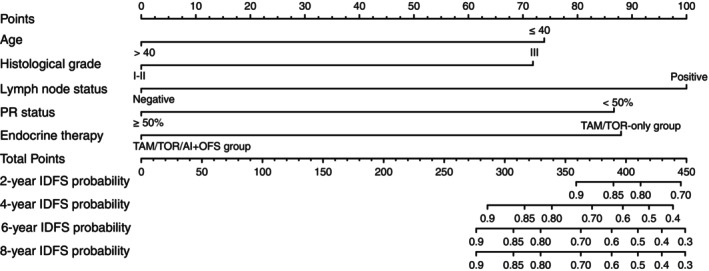
A nomogram was used to predict IDFS in premenopausal HR+/HER2+ early‐stage breast cancer patients who received trastuzumab. AI, aromatase inhibitor; IDFS, invasive disease‐free survival; OFS, ovarian function suppression; PR, progesterone receptor; TAM, tamoxifen; TOR, toremifene.

Univariate and multivariate Cox regression showed that age, histological grade, PR status, lymph node status, and OFS treatment were independent prognostic factors affecting IDFS in HR+/HER2+ early‐stage premenopausal breast cancer patients treated with trastuzumab (Table 2). The Kaplan–Meier survival curves of IDFS in different subgroups of patients are shown in Figures 1 and 3. Based on the multivariate Cox analysis of clinical‐pathological characteristics, we established a prognostic model of IDFS for these patients, demonstrated by a nomogram (Figure 4).

**TABLE 2:** Univariate and multivariate Cox regression analyses.

Characteristics	Univariate analysis		Multivariate analysis	
	HR (95% CI)	*p*‐value	HR (95% CI)	*p*‐value
Age/year				
≤40	Reference		Reference	
>40	0.507 (0.213–1.205)	0.124	0.352 (0.140–0.885)	0.026
Histological grade				
I–II	Reference		Reference	
III	2.783 (1.077–7.190)	0.035	2.759 (1.051–7.238)	0.039
Tumor size/cm				
≤2	Reference			
>2	1.025 (0.434–2.422)	0.955		
Lymph node status				
Negative	Reference		Reference	
Positive	2.828 (1.034–7.731)	0.043	4.107 (1.423–11.853)	0.009
Ki67				
<30%	Reference			
≥30%	2.052 (0.593–7.105)	0.257		
ER status				
<50%	Reference			
≥50%	0.590 (0.227–1.536)	0.280		
PR status				
<50%	Reference		Reference	
≥50%	0.239 (0.093–0.616)	0.003	0.294 (0.112–0.770)	0.013
LVI				
Negative	Reference			
Positive	1.589 (0.613–4.121)	0.341		
Surgery				
Lumpectomy	Reference			
Mastectomy	0.887 (0.321–2.447)	0.817		
Chemotherapy regimen				
Non‐anthracycline	Reference			
Anthracycline‐based	0.901 (0.346–2.348)	0.831		
Endocrine therapy				
TAM/TOR‐only	Reference		Reference	
TAM/TOR/AI+OFS	0.406 (0.149–1.110)	0.079	0.289 (0.100–0.835)	0.022

Abbreviations: CI, confidence interval; ER, estrogen receptor; HR, hazard ratio; LVI, lymphovascular invasion; OFS, ovarian function suppression; PR, progesterone receptor; TAM, tamoxifen; TOR, toremifene.

## DISCUSSION

The efficacy of OFS in premenopausal patients with HR+/HER2+ early‐stage breast cancer is still controversial. Some guidelines propose HER2 positivity alone as a risk factor and indicate that all HR+/HER2+ patients should receive OFS during endocrine therapy,^9^, ^10^ whereas others do not.^11^, ^12^ In addition, the vast majority of HR+/HER2+ breast cancer patients have received trastuzumab, and over half have been treated with anthracyclines; both of these therapies have some degree of cardiotoxicity, which may further be enhanced by OFS drugs. Therefore, it is important to carefully select HER2+/HR+ breast cancer patients who will actually benefit from OFS treatment. Based on previous studies and clinical practice, we conducted a retrospective review of premenopausal patients with HR+/HER2+ early‐stage breast cancer treated with trastuzumab to investigate the population that may benefit from the addition of OFS and the prognostic value of clinicopathological factors. To our knowledge, our study represents the largest study conducted in this specific population to investigate the efficacy of OFS, and the derivation of clinical data from multiple centers and the follow‐up period of 8.21 years lend confidence to the results. Notably, all patients were treated with trastuzumab in this study, which not only conforms to current practice in clinical treatment but also compensates for the shortcomings of previous studies and reduces the interference of confounding factors.

In our study, the average age of the patients was 42 years, 40.7% of patients were 40 years of age or younger, 47.4% were histological grade III, 53.4% had a tumor size of more than 2 cm, 54.2% were node‐positive, and 99.2% received adjuvant chemotherapy. These clinical features are similar to those of the ASTRRA study and riskier than those of the SOFT study. The ASTRRA study found no benefit of OFS in the HR+/HER2+ subgroup at either the 5‐ or 8‐year follow‐up, while the SOFT study showed the benefit of OFS in the HR+/HER2+ subgroup after the 5‐year follow‐up, which is consistent with the results of this study. However, limited by the era, only 61% received trastuzumab adjuvant treatment in the SOFT study, while trastuzumab treatment information related to the ASTRRA study is unknown. Therefore, this study better fills this gap, suggesting that in the context of the widespread use of trastuzumab, HER2 positivity alone can still be regarded as a risk factor for premenopausal HR+ breast cancer, and all HR+/HER2+ patients are recommended to accept endocrine therapy including OFS. Furthermore, the benefit of IDFS was more significant in the lymph node‐positive subgroup and age ≤40 years subgroup, further confirming the necessity of OFS treatment in premenopausal patients with intermediate‐to‐high‐risk HR+ breast cancer.

In previous studies, the SOFT study^3^, ^13^, ^14^, ^15^ established the role of OFS in adjuvant endocrine therapy among premenopausal patients with intermediate‐to‐high‐risk HR+ breast cancer. This study enrolled 2033 premenopausal patients with HR+ breast cancer, 236 (11.6%) with HR+/HER2+, and 141 (61%) with trastuzumab adjuvant therapy. At the 5‐year follow‐up, this study showed no benefit of OFS treatment in the overall population; the study performed well in the HR+/HER2+ subgroup, finding that the addition of OFS significantly reduced the risk of recurrence (HR = 0.42; 95% CI: 0.22–0.80), while poor performance in the HR+/HER2‐ subgroup showed no benefit (HR = 0.88; 95% CI: 0.69–1.13). Thereafter, the 8‐ and 12‐year follow‐up results showed benefits in the overall population, and the HR+/HER2+ subgroup performed better than the HR+/HER2‐ subgroup (8‐year HR: 0.41 vs. 0.83; 12‐year HR: 0.50 vs. 0.86). These results suggest that HER2 status affects the benefit of OFS treatment. Interestingly, among 141 patients treated with trastuzumab, the addition of OFS reduced the risk of recurrence by 39% (HR = 0.61; 95% CI: 0.99–0.95), which was a superior result compared to that of patients who did not receive trastuzumab, suggesting that whether treated with trastuzumab affected the benefit of OFS. In conclusion, the in‐depth analysis of the SOFT study suggests that HER2 status and trastuzumab adjuvant treatment influence the benefit of OFS treatment, and it is necessary to conduct research on premenopausal patients with HR+/HER2+ early‐stage breast cancer who received trastuzumab. In the ASTRRA study,^4^, ^16^ 1282 patients with premenopausal HR+ breast cancer who received chemotherapy were enrolled, including 176 (13.7%) HR+/HER2+ premenopausal breast cancer patients with unknown trastuzumab treatment status. No significant benefit was found from OFS treatment in the HR+/HER2+ subgroup at either the 5‐ or 8‐year follow‐up. Inconsistent with the SOFT study, treatment in the HR+/HER2+ subgroup of the ASTRRA study was less effective than that of the HR+/HER2‐ subgroup (5‐year HR: 0.71 vs. 0.59; 8‐year HR: 0.95 vs. 0.70), so there are still blind spots in OFS treatment for HR+/HER2+ patients. In addition, according to the ASTRRA study, the chemotherapy subgroup of the SOFT study and the meta‐analysis of large samples,^17^ authoritative guidelines all recommend OFS treatment for premenopausal HR+ breast cancer patients who need adjuvant chemotherapy. In our study, up to 99.2% of patients received adjuvant chemotherapy, which also demonstrated the benefit of OFS treatment. However, whether to administer adjuvant chemotherapy often changes with the update of evidence‐based data. Therefore, the question of whether to use chemotherapy as an indication is not rigorous, and more objective indicators should be used for evaluation.

Breast cancer exhibits considerable heterogeneity, so it is necessary to establish prognostic models to stratify these populations and administer tailored therapies. Currently, prognostic models based on multiple omics data, such as genes, digital pathological images, and clinicopathological features, play an increasingly critical role in the assessment of recurrence risk and the design of treatment regimens. For HR+/HER2− breast cancer, the 21‐gene assay and 70‐gene assay have been written into guidelines^18^ to predict prognosis and guide treatment decisions, and the HER2DX assay,^19^, ^20^ based on multigenomic data and clinicopathological features, is also being explored clinically. Our team also constructed a multimodal deep learning model for prognosis prediction in HER2+ breast cancer based on digital pathological images and clinicopathological features.^21^ However, HR+/HER2+ breast cancer possesses distinct molecular biological characteristics, and only a small number of patients with HR+/HER2+ breast cancer have been enrolled in previous models, making them less helpful for clinical decision‐making in these patients. In the present study, we identified age, histological grade, lymph node status, the expression level of PR, and OFS treatment as independent prognostic factors in these patients and established a prognostic model based on these clinicopathological characteristics. To our knowledge, this is the first time that a prognostic model has been developed specifically for HR+/HER2+ patients treated with trastuzumab. Hopefully, this model can be validated in an external cohort and improved by integrating multiomics data.

Given the research conditions, this study has the following limitations. First, the slow onset of endocrine therapy, the long course of treatment, and the late peak of recurrence associated with HR+ breast cancer suggest the importance of long‐term follow‐up for this type of study. Meanwhile, the SOFT and other studies also showed that the benefit of the addition of OFS increased with the extension of follow‐up time. This suggests that long‐term follow‐up is still important for this type of research. Although the median follow‐up in this study was 8.21 years, longer follow‐up is needed to fully assess treatment benefits. Second, to accommodate clinical practice and minimize potential confounding factors, all enrolled patients received trastuzumab, possibly resulting in the study not covering HR+/HER2+ patients with tumor sizes smaller than 0.5 cm. Finally, this study only provides real‐world evidence, which may still have follow‐up bias, selection bias, confounding factors and low‐quality data. Therefore, prospective studies in more centers and larger samples of HR+/HER2+ breast cancer patients are needed in the future.

## AUTHOR CONTRIBUTIONS

Study design: Jie Ju, Yi‐Xin Qi, and Peng Yuan. Data collection: Jie Ju, Song‐Lin Gao, Jia‐Yu Wang, Die Sang, Yi‐Kun Kang, Xue Wang, Jian Yue, and Yi‐Xin Qi. Data analysis: Jie Ju, Song‐Lin Gao, and You Shuai. Draft writing: Jie Ju and Peng Yuan. Final revision: Yi‐Xin Qi and Peng Yuan. All the authors have read and approved the final version of the manuscript and agreed with the order of presentation of the authors.

## FUNDING INFORMATION

National Natural Science Foundation of China (82172650); Chinese Academy of Medical Sciences Clinical Translational and Medical Research Fund (2022‐I2M‐C&T‐A‐014).

## CONFLICT OF INTEREST STATEMENT

All authors confirm and declare that they have no conflicts of interest.

## CONSENT FOR PUBLICATION

All authors agreed to publish this article.

## Supporting information


**Table S1.** The results of univariate Cox regression analyses in forest plot.

## References

[1] Sung H , Ferlay J , Siegel RL , Laversanne M , Soerjomataram I , Jemal A , et al. Global cancer statistics 2020: GLOBOCAN estimates of incidence and mortality worldwide for 36 cancers in 185 countries. CA Cancer J Clin. 2021;71(3):209–249. 33538338 10.3322/caac.21660

[2] Fan L , Strasser‐Weippl K , Li JJ , St Louis J , Finkelstein DM , Yu KD , et al. Breast cancer in China. Lancet Oncol. 2014;15(7):e279–e289. 24872111 10.1016/S1470-2045(13)70567-9

[3] Pagani O , Regan MM , Walley BA , Fleming GF , Colleoni M , Láng I , et al. Adjuvant exemestane with ovarian suppression in premenopausal breast cancer. N Engl J Med. 2014;371(2):107–118. 24881463 10.1056/NEJMoa1404037 PMC4175521

[4] Kim HA , Lee JW , Nam SJ , Park BW , Im SA , Lee ES , et al. Adding ovarian suppression to tamoxifen for premenopausal breast cancer: a randomized phase III trial. J Clin Oncol. 2020;38(5):434–443. 31518174 10.1200/JCO.19.00126

[5] Alataki A , Dowsett M . Human epidermal growth factor receptor‐2 and endocrine resistance in hormone‐dependent breast cancer. Endocr Relat Cancer. 2022;29(8):R105–R122. 35613334 10.1530/ERC-21-0293 PMC9254309

[6] Dieci MV , Guarneri V . Should triple‐positive breast cancer be recognized as a distinct subtype? Expert Rev Anticancer Ther. 2020;20(12):1011–1014. 33021124 10.1080/14737140.2020.1829484

[7] Ju J , Du F , Gao SL , et al. Combined analysis of receptor expression reflects inter‐and intra‐tumor heterogeneity in HR+/HER2+ breast cancer. Breast Cancer Res Treat. 2022;194(2):221–230. 35699854 10.1007/s10549-022-06629-w

[8] Giuliano AE , Connolly JL , Edge SB , Mittendorf EA , Rugo HS , Solin LJ , et al. Breast cancer‐major changes in the American joint committee on cancer eighth edition cancer staging manual. CA Cancer J Clin. 2017;67(4):290–303. 28294295 10.3322/caac.21393

[9] Chinese anti‐cancer association, Committee of Breast Cancer Society . Expert consensus on clinical applications of ovarian function suppression for Chinese women with early breast cancer 2021 CACA‐CBCS. China. Oncologia. 2022;32(2):177–190.

[10] The Society of Breast Cancer China Anti‐cancer Association . Guidelines for breast cancer diagnosis and treatment by China Anti‐cancer Association (2021 edition). China Oncologia. 2021;31(10):954–1040.

[11] Burstein HJ , Lacchetti C , Anderson H , Buchholz TA , Davidson NE , Gelmon KE , et al. Adjuvant endocrine therapy for women with hormone receptor‐positive breast cancer: American Society of Clinical Oncology clinical practice guideline update on ovarian suppression. J Clin Oncol. 2016;34(14):1689–1701. 26884586 10.1200/JCO.2015.65.9573

[12] Thomssen C , Balic M , Harbeck N , Gnant M . St. Gallen/Vienna 2021: a brief summary of the consensus discussion on customizing therapies for women with early breast cancer. Breast Care (Basel). 2021;16(2):135–143. 34002112 10.1159/000516114 PMC8089428

[13] Francis PA , Regan MM , Fleming GF , Láng I , Ciruelos E , Bellet M , et al. Adjuvant ovarian suppression in premenopausal breast cancer. N Engl J Med. 2015;372(5):436–446. 25495490 10.1056/NEJMoa1412379 PMC4341825

[14] Francis PA , Pagani O , Fleming GF , Walley BA , Colleoni M , Láng I , et al. Tailoring adjuvant endocrine therapy for premenopausal breast cancer. N Engl J Med. 2018;379(2):122–137. 29863451 10.1056/NEJMoa1803164 PMC6193457

[15] Francis PA , Fleming GF , Láng I , Ciruelos EM , Bonnefoi HR , Bellet M , et al. Adjuvant endocrine therapy in premenopausal breast cancer: 12‐year results from SOFT. J Clin Oncol. 2023;41(7):1370–1375. 36493334 10.1200/JCO.22.01065 PMC10419521

[16] Baek SY , Noh WC , Ahn SH , Kim HA , Ryu JM , Kim SI , et al. Adding ovarian suppression to tamoxifen for premenopausal women with hormone receptor‐positive breast cancer after chemotherapy: an 8‐year follow‐up of the ASTRRA trial. J Clin Oncol. 2023;41:4871. 10.1200/JCO.23.00557 37607321

[17] Bui KT , Willson ML , Goel S , Beith J , Goodwin A . Ovarian suppression for adjuvant treatment of hormone receptor‐positive early breast cancer. Cochrane Database Syst Rev. 2020;3(3):CD013538. 32141074 10.1002/14651858.CD013538 PMC7059882

[18] Bevers TB , Niell BL , Baker JL , Bennett DL , Bonaccio E , Camp MS , et al. NCCN guidelines® insights: breast cancer screening and diagnosis, version 1.2023. J Natl Compr Canc Netw. 2023;21(9):900–909. 37673117 10.6004/jnccn.2023.0046

[19] Denkert C , Wienert S , Poterie A , Loibl S , Budczies J , Badve S , et al. Standardized evaluation of tumor‐infiltrating lymphocytes in breast cancer: results of the ring studies of the international immuno‐oncology biomarker working group. Mod Pathol. 2016;29(10):1155–1164. 27363491 10.1038/modpathol.2016.109

[20] Prat A , Guarneri V , Pascual T , Brasó‐Maristany F , Sanfeliu E , Paré L , et al. Development and validation of the new HER2DX assay for predicting pathological response and survival outcome in early‐stage HER2‐positive breast cancer. EBioMedicine. 2022;75:103801. 34990895 10.1016/j.ebiom.2021.103801 PMC8741424

[21] Yang J , Ju J , Guo L , Ji B , Shi S , Yang Z , et al. Prediction of HER2‐positive breast cancer recurrence and metastasis risk from histopathological images and clinical information via multimodal deep learning. Comput Struct Biotechnol J. 2021;20:333–342. 35035786 10.1016/j.csbj.2021.12.028 PMC8733169

