# Supplementary material for: Prognostic factors and benefit populations of ovarian function suppression in premenopausal HR+/HER2+ early‐stage breast cancer patients who received trastuzumab: Evidence from a real‐world study with long‐term follow‐up
Source: Thorac Cancer. 2024 Jan 7;15(6):439–47. doi: 10.1111/1759-7714.15211 (PMC10883855; doi:10.1111/1759-7714.15211)
Supplement: Supplementary file 1 — Table S1. The results of univariate Cox regression analyses in forest plot. [file TCA-15-439-s001.docx]

**Supplementary table**

**Supplementary Table 1.** The results of univariate Cox regression analyses in forest plot.

| Characteristics | Total | TAM/TOR-only group | TAM/TOR/AI+OFS group | HR（95% CI） | P Value |
| --- | --- | --- | --- | --- | --- |
| Overall | 253 | 141(55.7%) | 112(44.3%) | 0.406(0.149-1.110) | 0.079 |
| Age/year |  |  |  |  |  |
| ≤ 40 | 103 | 47(45.6%) | 56(54.4%) | 0.272(0.074-1.007) | 0.051 |
| > 40 | 150 | 94(62.7%) | 56(37.3%) | 0.582(0.117-2.888) | 0.508 |
| Histological grade |  |  |  |  |  |
| I-II | 133 | 75(56.4%) | 58(43.6%) | 0.651(0.119-3.557) | 0.621 |
| III | 120 | 66(55.0%) | 54(45.0%) | 0.339(0.094-1.218) | 0.097 |
| Tumor size/cm |  |  |  |  |  |
| ≤ 2 | 118 | 74(62.7%) | 44(37.3%) | 0.566(0.116-2.776) | 0.483 |
| > 2 | 135 | 67(49.6%) | 68(50.4%) | 0.364(0.097-1.373) | 0.136 |
| Lymph node status |  |  |  |  |  |
| Negative | 116 | 84(72.4%) | 32(27.6%) | 1.981(0.327-11.979) | 0.457 |
| Positive | 137 | 57(41.6%) | 80(58.4%) | 0.153(0.043-0.537) | 0.003 |
| Ki67 |  |  |  |  |  |
| < 30% | 59 | 37(62.7%) | 22(37.3%) | 0.025(0.000-524.260) | 0.467 |
| ≥ 30% | 194 | 104(53.6%) | 90(46.4%) | 0.456(0.162-1.282) | 0.137 |
| ER status |  |  |  |  |  |
| < 50% | 52 | 39(75.0%) | 13(25.0%) | 0.758(0.085-6.784) | 0.804 |
| ≥ 50% | 201 | 102(50.7%) | 99(49.3%) | 0.394(0.125-1.240) | 0.111 |
| PR status |  |  |  |  |  |
| < 50% | 99 | 62(62.6%) | 37(37.4%) | 0.318(0.070-1.448) | 0.139 |
| ≥ 50% | 154 | 79(51.3%) | 75(48.7%) | 1.061(0.214-5.258) | 0.942 |
| LVI |  |  |  |  |  |
| Negative | 145 | 93(64.1%) | 52(35.9%) | 0.229(0.029-1.839) | 0.166 |
| Positive | 86 | 34(39.5%) | 52(60.5%) | 0.545(0.146-2.034) | 0.366 |

Abbreviations: LVI, lymphovascular invasion; ER, estrogen receptor; PR, progesterone receptor; OFS, ovarian function suppression; TAM, tamoxifen; TOR, toremifene; HR, hazard ratio; CI, confidence interval.
